# POSERS: A Steganography-Driven Molecular Tagging System Using Randomized DNA Sequences for Secure Authentication

**DOI:** 10.1021/acsomega.5c07126

**Published:** 2025-10-23

**Authors:** Ali Tafazoli Yazdi, Peter Nejjar, Lena Hochrein

**Affiliations:** † 26583 University of Potsdam, Institute of Biochemistry and Biology, Faculty of Science, Potsdam 14476, Germany; ‡ 26583 University of Potsdam, Institute of Mathematics, Faculty of Science, Potsdam 14476, Germany

## Abstract

Counterfeiting remains a major threat across multiple industries, causing substantial financial losses and health-related risks. DNA-based molecular tagging has emerged as a promising anticounterfeiting strategy, but existing methods rely on predefined DNA sequences, making them increasingly vulnerable to replication as sequencing and synthesis technologies advance. To address these limitations, we introduce POSERS (Position-Oriented Scattering of Elements among a Randomized Sequence), a steganographic DNA tagging system that encodes product-specific constraints into highly diverse, randomized DNA libraries. While the underlying design is adaptable to other molecular or material carriers, DNA offers a particularly attractive option due to its chemical stability, low synthesis cost, and proven compatibility with materials such as inks, polymers, and coatings. Unlike previous methods that use fixed tags, POSERS enables customizable design complexity and offers resilience against replication attempts. Each product in a batch receives a unique DNA library generated in a single synthesis step, allowing for cost-effective, large-scale deployment. The security of POSERS is mathematically validated and experimentally confirmed using next-generation sequencing, combined with complementary authentication tests that detect both unauthorized sequence combinations and insufficient diversity. POSERS consistently distinguishes genuine tags from forgeries, even under conservative assumptions about an attacker’s knowledge and capabilities. Our approach prevents both PCR-based copying and reverse-engineering of tag designs. With its customizable design parameters, minimal DNA input requirements, and demonstrated robustness against current and emerging attacks, POSERS establishes a new standard for secure molecular tagging and offers a scalable, future-proof solution for high-risk products.

## Graphical abstract

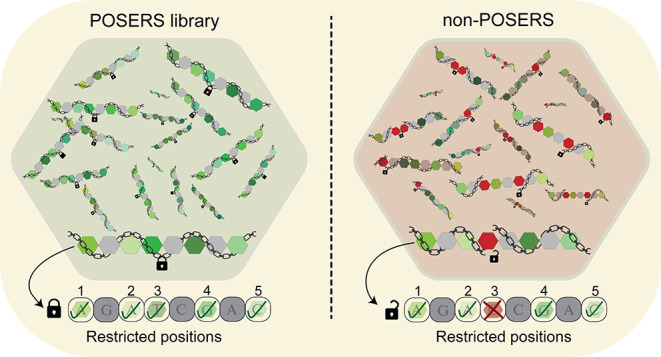

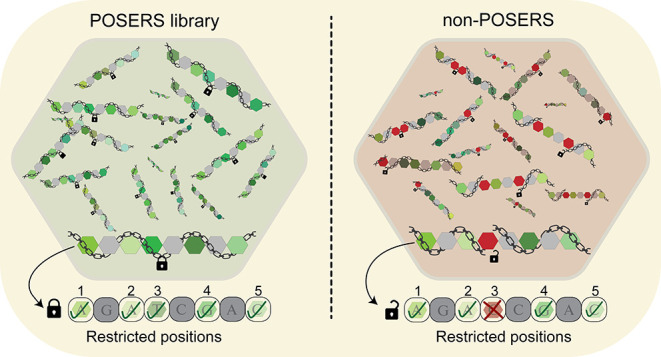

## Introduction

Steganography is the practice of concealing the existence of a message within seemingly ordinary data to maintain the confidentiality of the message. The origins of steganography can be traced back to 440 BC, where it was employed to hide messages on writing tablets. Today, steganography is utilized in the transmission of digital multimedia data, including images, audio, and video. The decreasing cost of DNA synthesis and sequencing has expanded the use of DNA as a medium for steganography, enabling new applications in secure information embedding and molecular data storage.^1,2^

This development benefits another area that deals with the use of DNA as a means of labeling products to securely track and authenticate them. Considering that counterfeiting occurs in many different industrial sectors, such as clothing, footwear, luxury items, vehicles but also pharmaceutical industry it quickly becomes clear that this not only leads to a financial loss for the industry, but also to a health risk for the end consumer.^3–5^ Thus, various approaches to DNA-based molecular tagging have been reported in recent years to increase anticounterfeiting protection compared to traditional means such as UPC barcodes, QR codes, RFID or watermarks.^6^

DNA-based molecular labeling uses DNA sequences to mark high-value or security sensitive items —visible or invisible—so that they can be identified, tracked and protected from tampering. The available methods essentially differ in the types of DNA molecules and the readout of DNA sequences used to proof authenticity of the analyzed sequence. Some systems use hybridization-based fluorescence readouts, where a correctly matched reporter strand triggers a visible signal.^7^

Other approaches, such as the Porcupine system, employ synthetic “molbits” identified by Oxford Nanopore sequencing within minutes, bypassing the need for full basecalling by interpreting unique signal patterns.^8^ Luescher et al. introduced chemical unclonable functions (CUFs) based on operable random DNA pools.^9^ These CUFs utilize pools of up to 10^10^ unique sequences that respond to specific PCR inputs with distinctive sequence outputs, validated via next-generation or Sanger sequencing.^9,10^

While current methods for DNA-based molecular tagging differ in how they encode and detect DNA sequences, they all rely on a fixed set of DNA sequences, shared across product batches. These systems are considered forgery-resistant due to the current limitations in DNA synthesis and sequencing technologies. However, motivated forgers can exploit existing molecular biology techniques to identify and replicate these tags. As DNA sequencing and synthesis technologies continue to improve rapidly, such methods may become increasingly vulnerable. This raises the important question of how we can design a DNA-based tagging system that guarantees long-term, cost-effective security, even as sequencing and synthesis technologies continue to evolve.

Here, we introduce POSERS (**p**osition-**o**riented **s**cattering of **e**lements among a **r**andomized **s**equence), a steganographic tagging system embedded within randomized DNA libraries. POSERS overcomes the limitations of traditional tagging systems by encoding product-specific constraints within highly diverse sequence pools. Its design allows customizable complexity levels based on application needs and ensures resilience against future advancements in DNA sequencing and synthesis. We mathematically validate the security of this approach and demonstrate its practical applicability through next-generation sequencing and authentication tests that reliably distinguish genuine POSERS tags from forged ones. To demonstrate real-world applicability, we implemented and tested POSERS on a paper substrate, validating its compatibility with commonly used carrier materials.

## Results

### DNA Protection: The Secure Design of POSERS

The POSERS system presents a steganographic approach to designing and producing DNA-based molecular tags, utilizing DNA libraries that contain a large number (typically millions) of unique sequences, each embedding a specific forgery-proof design. This design prevents a forger from deciphering or copying the sequence, while still allowing the designer to reliably authenticate the DNA library. In simple terms, it works by selectively excluding certain combinations of nucleotides within an otherwise randomized DNA sequence.

Depending on the desired security level of the customer (e.g., related to product lifespan, batch size, technological advancements or anticipated attack sophistication), we define a POSERS design tailored to a specific product batch. Each design specifies the characteristics of the DNA sequences of one batch, while every individual product in the batch is labeled with a unique set of sequences following the same POSERS design.

The design of POSERS libraries consists of three main steps (Figure 1):

**Figure 1:**
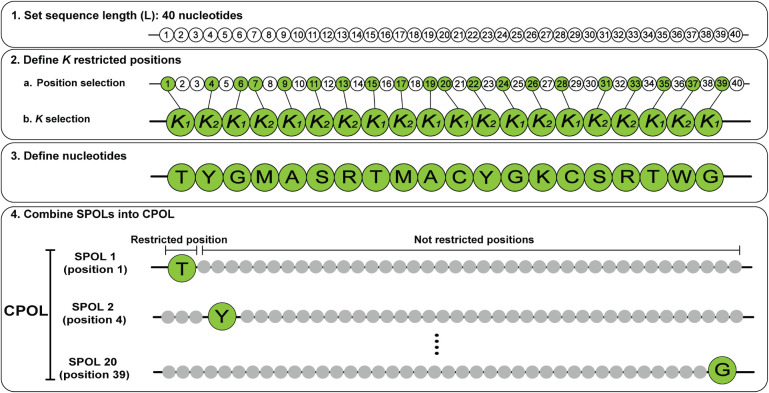
POSERS design parameters and synthesis of the suggested single position oligo libraries (SPOLs) and the combined positions oligo library (CPOL). In step 1, the length of DNA strands is defined to be 40 nucleotides (illustrated as circles). In step 2, the number of restricted positions *K* is defined to be 20 and positions are chosen in a (green circles). The 20 restricted positions are defined to hold either one nucleotide (***K**_1_*) or two nucleotides (***K**_2_*) in b. In step 3, the specific nucleotides allowed at each restricted position are selected (IUPAC codes: Y= C/T; M = A/C; S = C/G; *R* = A/G; K = T/G; W = A/T). In step 4, for each restricted position, a unique SPOL library is synthesized in which the picked position (green circle) contains only the assigned nucleotide(s), while all other positions (gray circles) are unrestricted (all four nucleotides). All 20 SPOLs generated in the pool synthesis are combined to form the CPOL.

(1) **Set sequence length (** *L* **):** Each DNA strand in the design has *L* nucleotides. Nucleotides are the basic building blocks of DNA, each carrying one of four bases: A, T, C, or G.

(2) **Define** ***K*** **restricted positions:** Along the DNA strand, *K* positions are chosen where the choice of nucleotide is intentionally restricted.

• *K*_1_ positions: only one allowed nucleotide

• *K*_2_ positions: two allowed nucleotides

• *K*_3_ positions: three allowed nucleotides

Importantly, the locations of these restricted positions, as well as the specific type of each restricted position (*K*_1_, *K*_2_ or *K*_3_) are confidential and known only to the designer. The number *K* directly determines the number of single position oligo libraries (SPOLs) in the design, as each SPOL corresponds to one restricted position.

(3) **Define nucleotides for each restricted position:** For each restricted position, choose the exact nucleotide(s) allowed according to its type (*K*_1_, *K*_2_ or *K*_3_). The remaining positions are randomly filled with an equal distribution of all four nucleotides.

(4) **Combine SPOLs into the CPOL:** All SPOLs are mixed in equal amounts to form the combined positions oligo library (CPOL), which constitutes the complete POSERS DNA library for one product batch. The total design includes *K* SPOLs (*K*= *K*_1_+*K*_2_+*K*_3_ ).

A key practical advantage of POSERS is that POSERS DNA libraries can be produced using standard oligonucleotide pool synthesis^11^ (Figure 1), which enables the generation of thousands of unique DNA strands in a single, cost-effective synthesis step. Rather than ordering each sequence individually, all variability is encoded directly into the synthesis design.

For experimental validation, we used single-stranded DNA sequences of a length (*L*) of 40 nucleotides, with ten *K*_1_ positions and ten *K*_2_ positions, giving 20 SPOLs in total. These were combined to form the CPOL. Importantly, in the CPOL, each restricted position is statistically concealed by mixing one SPOL containing the restriction with 19 SPOLs without that restriction, resulting in an overall uniform nucleotide distribution. However, missing nucleotide combinations between restricted positions remain detectable, allowing authentication. The CPOL can be used to tag either a single product (e.g., a high-value product with a long lifespan) or an entire batch of products, illustrating the flexibility and scalability of the POSERS framework.

### Exposing Counterfeits: Distinguishing Authentic and Nonauthentic Libraries

For the authentication of a certain product, which is tagged by a POSERS DNA library, DNA needs to be extracted from the product and sequenced via next generation sequencing (NGS). It is evident that any DNA sample that does not meet the defined POSERS design criteria — such as sequence length, restricted positions, or library composition —is immediately classified as invalid and rejected. However, if the sample exhibits the general characteristics of a POSERS design, we proceed to assess the authenticity of a tagged product by further analyzing the sequences of the CPOL.

First, we perform the sample combination test: a DNA sequence is considered as authentic, if it either belongs to one of the *K* SPOLs, or does not include any restricted combination. Otherwise, a DNA sequence is considered nonauthentic. The sample combination test relies on finding restricted combinations in a nonauthentic tag.

To determine the sample size of the sample combination test, we need to first calculate the probability of encountering a restricted combination in a non-POSERS DNA library. To do so, we use the example of a randomized library as a nonauthentic tag as it might represent the most probable scenario for a forged DNA tag. Since a POSERS design excludes certain combinations of nucleotides at restricted positions, the resulting DNA library is smaller compared to a fully randomized library without any restrictions. As a result, nonauthentic sequences will be included if a forger adds a randomized library instead of a POSERS library. A library is considered a forgery if the analysis yields a sufficiently high number of nonauthentic sequences. To determine the number of sequences that must be analyzed to reliably detect a forgery, we computed the absolute numbers and the proportion of nonauthentic and authentic sequences among all possible sequences that would result from a randomized library. The theoretical proportion of nonauthentic sequences, the missing rate *p*, equals:

1 $$p={\left(\frac{3}{4}\right)}^{K_{1}}{\left(\frac{1}{2}\right)}^{K_{2}}{\left(\frac{1}{4}\right)}^{K_{3}}$$

Since any sequence is either authentic or nonauthentic, the proportion of authentic sequences equals 1–*p*. Thus, among the 4^*L*^ possible sequences, there will be *p*4^*L*^ nonauthentic sequences, and (1–*p*)­4^L^ authentic sequences. For the exemplary POSERS design in this study, the missing rate *p* equals 5.4994*10^‑5^. Therefore, we calculate the number *n* of DNA sequences that need to be analyzed in a randomized library to ensure, with very high probability, the detection of a nonauthentic sequence.

2 $$n=\frac{\ln\left(\varepsilon \right)}{\ln\left(1-p\right)}$$

For the current CPOL, we choose the parameter ε, which represents the possibility to not detect a fully randomized library as a forgery, to be 0.00001%. In this way, the number of sequences equals *n*=2.5121*10^5^. Thus, we define *n* as the number of sequences the designer must test to ensure that the library can be reliably authenticated and distinguished from a randomly generated library. The mathematical verification of eqs 1 and 2 is provided in the Supporting Information.

DNA synthesis and sequencing may introduce errors and biases that deviate from the ideal mathematical assumptions.^12,13^ To ensure the secure authentication of a POSERS library, the errors of the experimental steps must be taken into account (Figure 2). For this purpose, an exemplary CPOL was synthesized by the company Integrated DNA Technologies (IDT) using oligo pool synthesis service. The transposase used for sequencing library preparation in this study has previously shown to remove approximately 50 bp from each end of a DNA sequence.^14^ To ensure a sufficient length for sequencing of the CPOL library, DNA sequences contain 40 nucleotides of the designed sequences flanked by 80 nucleotides of fixed constitutive sequences on both the 5′ and 3′ ends. As a negative control, the same library is ordered, but with the design replaced by 40 nucleotides of randomized sequence. The negative control is also generated using a pooled synthesis approach, alongside a second pool sample in which the design is replaced by a synthetic constitutive sequence. The constitutive DNA sample was used solely as proof of successful sequencing sample preparation and was not included in further analysis. Both libraries are ordered as single-stranded and are converted to double-stranded libraries prior to testing. For sequencing, we used the Illumina sequencing platform 2*150 with a PCR-free kit to minimize the effect of PCR duplication on the original sample. The sequencing was performed as a paired-end run. However, since a single read covers the full length of the design, the analysis is based on single reads. Twenty-five nanograms of both the double-stranded CPOL and the double-stranded control sample were used for sequencing, as this is the minimum sample quantity recommended by the PCR-free kit protocol.

**Figure 2:**
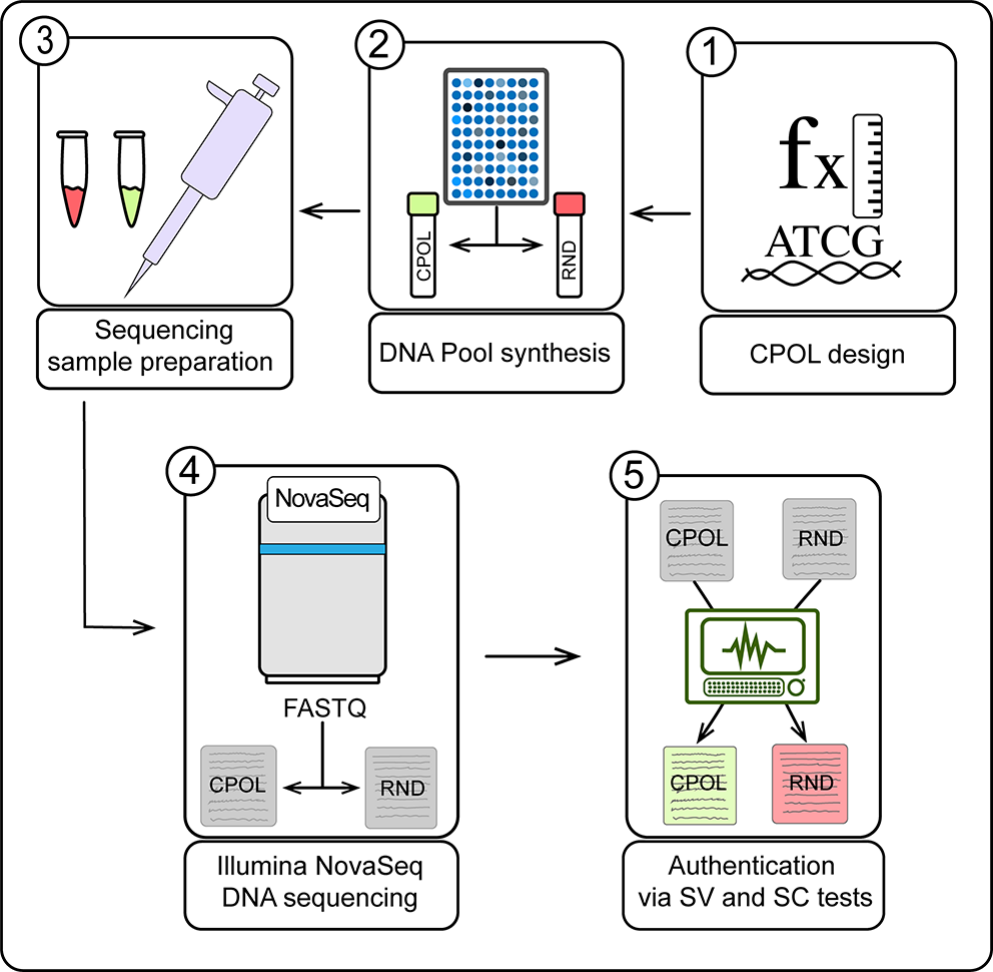
Schematic overview of the experimental design for generating and authenticating a POSERS DNA library. 1) A POSERS design is selected considering factors including the length of DNA sequences (L), the number of restricted positions (*K*), and the nucleotides allowed in each of the *K* positions. 2) The combined positions oligo library (CPOL) sample and a control consisting of a fully random library (RND) are synthesized through separate DNA pool syntheses. 3) Samples are prepared for Illumina sequencing by double-stranding and library preparation using Illumina DNA PCR-Free prep. 4) The sequencing is performed on the Illumina NovaSeq platform, generating FASTQ results. 5) The FASTQ results from the CPOL and random samples are checked using the authentication program. After performing the sample combination (SC) and sample variety (SV) tests, the CPOL sample is approved, while the random sample is rejected.

The raw data obtained from the sequencing experiment was analyzed by a custom program (see Methods) to filter out the duplicated reads and reads that do not follow the CPOL design with respect to the length and correct primer binding sites. This resulted in 1,029,652 unique sequences from the CPOL sample and 468,156 sequences from the random control sample. The POSERS authentication program subsequently finds all the restricted combinations in these sequences according to the design (see Methods). As expected, the program did not detect any forbidden combinations in the original sample. In contrast, 29 forbidden combinations were identified in the randomized control sample (Table S1). This means that the probability of a sequence being forbidden in a library of completely random sequences can be estimated as 29 divided by 468,156, which equals 6,194516*10^‑5^. Since the estimated probability is close to, and even slightly higher than the mathematically calculated probability (*p*= 5.4994*10^‑5^), it can be concluded that experimental errors from DNA synthesis and sequencing do not negatively impact the effectiveness of the sample combination test in identifying restricted combinations. Furthermore, the original CPOL could be securely distinguished from the randomized control sample.

Second, to ensure that the sequencing result of the test sample accurately represents the complexity of the original sample, we recommend conducting the sample variety test performed by the POSERS authentication program. Here, we consider a sample as authentic if it follows the expected design variety and includes all allowed nucleotides in each SPOL. By isolating sequences from the sequencing data that can only result from a single SPOL, we can analyze whether all allowed nucleotides are present at the restricted position. This is achieved by selecting sequences in which all positions, except for the position of interest, contain a restricted nucleotide. This ensures that each restricted position includes all allowed nucleotides and reveals a scenario in which a forger correctly predicts the position of a restriction but not the type of restriction (*K*_1_ instead of *K*_2_ or *K*_3_). For example, a sequence can be clearly assigned to SPOL 22 if all other positions contain nucleotides which are excluded in their respective SPOL. By examining all positions in the CPOL sample, we confirmed that all allowed nucleotides are present at the restricted positions where SPOL specific sequences are found (Table S2). This test therefore serves as proof for the diversity of the sample. Thus, a DNA tag imbedded within a POSERS design can be reliably authenticated by a combination of the sample combination and variety tests.

### Breaking the Design: How Forgers Might Attack the Library

If forgers attempt to counterfeit products tagged by POSERS, they can pursue two strategies: either multiplying the existing DNA library or identifying the design of the POSERS tags to synthesize a forged DNA library.

### First Angle of Attack: Copying the Sample

The first approach a forger could take to generate authentic DNA sequences is to gain access to a number of authentic products, denoted as *R*. As each product is tagged with at least *n* sequences, the forger has access to R*n authentic sequences, which they may proceed to use for copying the authentic sequences. Given the current state of DNA synthesis and sequencing, there are two options available for copying the DNA sample:

#### (1) Synthesizing a New Copy of Isolated and Identified Sequences

The first approach a forger could take is to isolate DNA from the accessible original product and perform a sequencing experiment to identify as many authentic sequences as possible. Subsequently, the sequencing data can be used to resynthesize the authentic sequences. However, the built-in variety of the design guarantees that synthesizing such a large number of sequences (at the moment >100,000 unique sequences per product) individually remains entirely impractical. While the cost of DNA synthesis decreases overtime, the POSERS design allows for an increase in sequence variety as needed, ensuring that this approach remains unfeasible. Remarkably, if the same library of synthesized fragments will be applied on several products, the authentication test will identify the forgery by tracking duplicate sequences.

#### (2) Duplicating an Original POSERS Library by Amplification

The second approach to copying a POSERS library involves amplifying DNA isolated from the original product using PCR. An important feature of POSERS is that any amplification attempt, unless it is 100% efficient, can be traced and detected. Accordingly, the POSERS design incorporates technical hurdles that restrict high-efficiency PCR amplification of the POSERS library: First, the POSERS library will be designed as single-stranded DNA sequences with only a single primer binding site. This would allow the designer who knows the primer binding site to convert the single-stranded library efficiently into a double-stranded DNA library but prevents the forger from directly amplifying the library using PCR. Additionally, providing the sample as single-stranded DNA further complicates the identification of the primer binding sequence which is necessary to generate a double-stranded library. To bypass this restriction and amplify the original library, a forger would need to perform multiple enzymatic steps: attaching a single-stranded oligo tag carrying another primer binding site to the other end of the single-stranded POSERS sequence,^15^ PCR amplifying or subcloning the resulting DNA fragments and finally removing the added oligo tags from all DNA sequences without leaving any detectable scar sequences. This process is not only highly resource-intensive, but the multiple enzymatic reactions required for the attachment and subsequent removal of primer binding sites are also likely to leave detectable residues at the ends of the POSERS design and operate with very low efficiency. As a result, the forged library exhibits low diversity and contains unwanted sequence residues that can be detected during authentication. This makes it clear that even if a forger would take the effort and still copy the POSERS library by PCR, the authentication program will identify this copy due to a lower variety of sequences and detectable residues from enzymatic reactions.

Furthermore, the presence of duplicated sequences could serve as an additional measure for authenticating a POSERS tag, given that a forged PCR-amplified sample would exhibit a higher number of duplicated sequences due to its lower diversity. Since only a fraction of the theoretically possible sequences from the total POSERS design is synthesized, we expect an authentic POSERS tag to be free of duplicates. However, when tested on a single product, this approach proved unsuitable because optical duplicates, an artifact of Illumina sequencing,^16^ obscured the detection of true duplicates (Figure S1). Selecting alternative Illumina sequencers or exploring alternative NGS platforms such as Nanopore and PacBio could enable the identification of duplicates as an additional measure for detecting a copied library, along with testing for the lower diversity and sequence residues.

Remarkably, the challenge of detecting duplicates arises only when the forged PCR-amplified library is applied to a single product. If the forger uses the PCR-amplified tag across multiple products, these products are expected to undergo independent sequencing runs. In this scenario, no optical duplicates should be present between runs. Therefore, any duplicated sequences identified across different sequencing runs can be attributed to PCR amplification, indicating that the tag was forged. Consequently, all products sharing the duplicated sequences can be classified as counterfeit.

### Second Angle of Attack: Finding the Design

The second angle of attack is to find the specific design, meaning the number and kind of restricted positions, by sequencing an authentic DNA library. This approach would be the only way for the forger to synthesize a POSERS library in a cost-effective manner. To do so a forger has two general options:

#### (1) Analyzing Distribution Differences of Nucleotides at Individual Positions

Forgers could try to detect irregularities in the nucleotide distribution at certain positions of the DNA sequence caused by the implementation of the POSERS design. The implementation of a POSERS design in a nucleotide library results in a slight bias in nucleotide distribution at the picked restricted positions, while positions without restrictions have no bias with an expected theoretical proportion of 25% for each of the four nucleotides. However, at a position which has been picked, the expected proportion of an allowed letter is different. Namely, at a position where *i* = 1, 2, 3 letters are allowed in the design, the expected theoretical proportion of an allowed letter is

3 $$\frac{1-1/K}{4}+\frac{1}{\mathrm{iK}}$$

whereas the not-allowed letters have an expected proportion of

4 $$\frac{1-1/K}{4}$$

The mathematical verification of eqs 3 and 4 is provided in the Supporting Information. Applied to our POSERS design with 20 restricted positions and two allowed nucleotides, a deviation of 1.25% from the average results in a proportion of 26.25% for the allowed nucleotides and 23.75% for the not-allowed nucleotides. For a scenario with one allowed nucleotide and 20 restricted positions, a deviation of 3,75% leads to a proportion of 28.75% for the allowed nucleotides and 23.75% for the not-allowed nucleotides.

Based on the given mathematical assumption, we proceed with predicting how a forger might attempt to reconstruct the CPOL design. We assume the forger was able to successfully sequence the original CPOL from an authentic product and attempts to predict a design based on the assumption that the design has 20 restricted positions.

We calculate the average nucleotide distribution across all 40 positions in all reads based on the FASTQ data, resulting in the following distribution: A: 20.68%, T: 29.57%, C: 18.83%, and G: 30.93%. We designate positions where the nucleotide distribution exceeds the average by 1.25% (deviation from average calculated for *K* = 20, *i* = 2) as *K* restricted positions (Table S3). If a forger attempted to predict the design using the suggested approach, their prediction would result in the pattern shown in Figure 3A. However, the forger would only be able to identify 17 restricted positions correctly, leading to nine incorrect predictions in the forged library. Before investigating how false predictions can be identified in the forged library, we first categorize the two different types of falsely predicted positions (Figure 3B):

**Figure 3:**
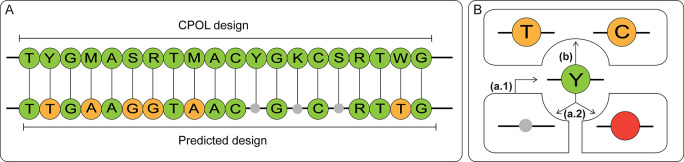
False design prediction from nucleotides distribution analysis. (A) The predicted design, based on the nucleotide distribution analysis, includes nine falsely predicted positions: three predictions of scenario (a.2) (gray circles) and six predictions of scenario (b) (yellow circles). (B) Type (a.1) prediction: an unrestricted position in the original design is considered as a position with restriction. Type (a.2) prediction: a restricted position (here Y = C or T, depicted as green circle) is not recognized (small gray circle) or the prediction for this position includes a forbidden nucleotide (red circle). Type (b) prediction: a restricted position was correctly predicted, but not all nucleotides allowed in this position are recognized.

Scenario (a.1): The first type of false prediction occurs when a position without restrictions is falsely predicted as a restricted position. This results in the synthesis of an additional SPOL by the forger introducing a restriction at a false position. Since all other positions besides this restricted position are filled with all four nucleotides, this introduces incorrect nucleotide combinations into the sample at positions that are restricted in the original design. In this way, this SPOL introduces all false combinations that a fully randomized library would also introduce.

Scenario (a.2): The second type of false prediction occurs when a restricted position in the original design is either not identified at all, or it is identified incorrectly and includes at least one nucleotide restricted from this position in the original design. In this scenario, the forger either excludes a SPOL from the CPOL design, resulting in a random distribution of all four nucleotides at this position, or includes a SPOL with at least one forbidden nucleotide included. Consequently, this position contains nucleotides that are excluded by the original design, leading to restricted combinations in the forged sample that do not exist in the authentic sample. The predicted design depicted in Figure 3A includes three examples of (a.2) predictions.

The introduction of restricted nucleotide combinations resulting from false predictions of type (a) can be identified by the sample combination test. However, the nonauthentic library with a certain number of correctly predicted positions incorporates fewer false combinations in comparison to the randomized library. This should be considered to adjust and increase the number of sequences *n* that needs to be analyzed, calculated from eq 2. As soon as a single SPOL with a type (a) prediction is present in a CPOL, we can compute the probability that a sequence from the CPOL is not authentic as $p^{\prime }\geq 2\;*\;\frac{p}{3K}$ . This means that the number of sequences we need to test from a POSERS library is calculated by multiplying the number *n* from eq 2 by $\frac{3K}{2}$ . For the suggested CPOL design, this means that the number of sequences to be tested increases from 2.5121*10^5^ sequences per library to 7.5363*10^6^ to ensure the detection of a type (a) prediction in a forged sample.

The downside of increasing the number of *n* is the higher cost associated with incorporating more DNA into the tag and the increased sequencing requirements. However, if necessary, adjusting *n* allows for maximizing the security level of authentication for the POSERS tag, ensuring that even false combinations introduced by a single type (a) prediction can be detected by the sample combination test.

Scenario (b): The third type of false prediction occurs when the forger correctly identifies the restricted position but fails to determine all allowed nucleotides at that position. For example, if a designed position allows two nucleotides, but the forger incorrectly only allows one nucleotide and thereby restricts one nucleotide. In this way, no false combinations are introduced into the library. However, all DNA sequences generated from the forged SPOL will lack the missing nucleotide(s) at that position. As a result, type (b) prediction can be detected by our authentication program using the sample variety test. The predicted design contains six predictions of scenario (b) (Figure 3A).

Therefore, unless the forger accurately predicts all restricted positions and all allowed nucleotides at these positions, the forged sample will either contain false predictions or limited diversity, which can be traced back by the POSERS authentication tests and used to distinguish it from the original sample. Furthermore, the variables during DNA synthesis, such as the composition of the nucleotide mixture and the techniques used for wobble oligo synthesis and DNA pooling synthesis, can vary from sample to sample, as they differ between synthesis companies. Therefore, a forger would need access to the specific DNA synthesis information for each sample of one batch to develop an optimal prediction strategy. Without detailed knowledge of the POSERS design and the exact synthesis method, it would be extremely difficult for a forger to develop a more precise or effective prediction method than the one demonstrated in this study. Furthermore, the POSERS designer can make prediction even more challenging by adjusting the ratio of individual SPOLs within the design. In the CPOL design presented here, it is assumed that all SPOLs are present in equal proportions within the sample. However, by modifying the DNA pooling after synthesis, the designer can alter the relative representation of individual SPOLs in the final CPOL, further complicating the prediction process. Finally, the designer can fine-tune the design parameters, such as increasing the number of restricted positions. This, in turn, raises the number of SPOLS, thereby minimizing deviation from the average calculated using eq 3 and making prediction significantly more difficult.

#### (2) Check the Combinations of Restricted Positions

The second strategy for deciphering the design involves analyzing all nucleotide combinations in the sample to identify abnormal restrictions, such as the absence of certain nucleotide combinations. When analyzing the nucleotide combinations, the key difference between the designer and the forger lies in the number of sequences that need to be examined. The designer knows the exact locations of restricted positions and which restrictions to check, significantly reducing the number of sequences that need to be analyzed. This number, defined as the lower threshold, is the minimum number of sequences that need to be applied on one product (*n* calculated from eq 2). In contrast, the forger lacks this information and must analyze a much larger number of sequences to identify the restrictions. This defines the upper threshold which is set by the number of sequences a forger would have to analyze to identify the POSERS design. Applying fewer sequences as this number per product ensures that the design remains secure and cannot be deciphered. While in a real scenario the forger would not have knowledge of all restricted combinations, we assume here that future methods could potentially generate this information. In that case, the forger could analyze the number of produced combinations only at the *K* picked restricted positions, e.g., by systematically going through all values for *K* and all the $\left(\begin{array}{l} L\\ K \end{array}\right)$ possible ways to pick *K* positions. In this way, when K reaches 20, the forger might realize that *p*4^*K*^ sequences are never produced and conclude that these combinations are forbidden. Since K<L, the forger needs access to fewer sequences to make this conclusion. Therefore, the number of DNA sequences at the upper threshold must be limited so that for the *K* restricted positions, no more than (1–*p*)­4^*K*^ combinations are possible. The simplest way to achieve this is to produce fewer than (1–*p*)­4*^K^* DNA sequences per design and in this way limit the amount of available DNA sequences, which will prevent a counterfeiter from finding the design. While we use the simplest approach here to define the upper threshold, mathematical justification supports the generation of even more sequences while still ensuring the safety of the design (Supporting Information).

The security resistance of the POSERS design against the two forgery attempts presented here demonstrates its effectiveness in generating a forgery-proof and copy-resistant DNA tag.

### Defining the Application: How Many Products Can Be Tagged with One POSERS Design

The defined upper and lower threshold is used to calculate the number of unique products that can be tagged with a single POSERS library following one design. For the CPOL design defined here, the lower threshold is calculated to be *n*=2.5121*10^5^. The number of products *P* is then constrained by the inequality *Pn*≤(1–*p*)­4*^K^*, such that

5 $$P\leq \frac{\left(1-p\right)4^{K}}{n}$$

In this way, the minimum number of products that can be tagged by the current CPOL is *P*=4.3766*10^6^. This demonstrates the potential of a single POSERS design to tag a large number of products before a design change becomes necessary. In addition to the mathematical calculations, these findings must be experimentally validated to assess their practical feasibility. This includes determining the minimum amount of DNA required for sequencing to reach the lower threshold of reads. Additionally, the impact of applying DNA to a product — a paper substrate — as well as the subsequent extraction process on the sequencing results needs to be examined. Therefore, we prepared seven dilutions of the double-stranded library, ranging from 0.01 ng to 25 ng. All samples were multiplexed and used in one sequencing run (sample 1–5, 7; see Methods section). The results indicate that using 5 ng of the CPOL can yield sufficient sequencing results to cover the 2.5121*10^5^ reads required for the authentication process (Figure 4A).

**Figure 4:**
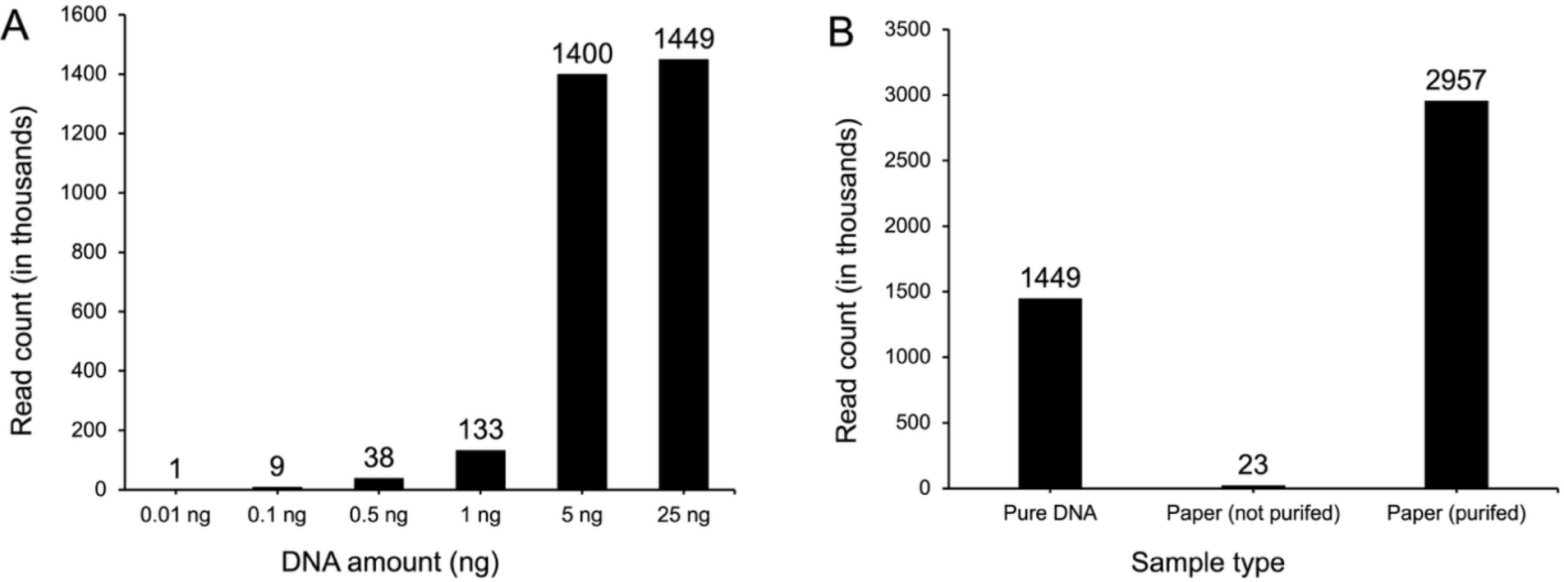
Experimental impact on DNA sequencing output. (A) The effect of DNA input on sequencing results, showing increasing sequencing output for samples of the combined positions oligo library (CPOL) with input amounts ranging from 0.01 ng to 25 ng. (B) The impact of applying the combined positions oligo library (CPOL) to paper and subsequent DNA purification on sequencing output, following DNA isolation from paper.

To investigate the effect of applying a POSERS library to a product, the DNA was applied to paper, extracted, and subsequently sequenced. Two samples were prepared for this test. In both cases, 25 ng of the DNA library was applied to filter paper. After drying for 24 h, the DNA was extracted by applying water to the paper and collecting the eluate. For one sample, the extracted DNA was directly used for sequencing preparation. For the other sample, the DNA was purified using a purification kit before proceeding with sequencing preparation (sample 7, 9, 10; see Methods section). The results showed that, unlike the nonpurified sample, the purified sample achieved a number of sequencing reads comparable to the untreated sample that was not applied to paper (Figure 4B). Interestingly, the purified DNA extracted from the paper yielded more than twice the sequencing output compared to 25 ng of the pure DNA library. However, this difference can be explained considering errors in sample preparation handling. These findings indicate that applying DNA to paper and subsequently extracting dried DNA does not considerably impact sequencing output, provided that the sample undergoes purification after isolation.

## Discussion

In this study, we introduced POSERS, a steganographic approach for generating DNA tags with unmatched security properties, including copy and forgery protection.

We mathematically demonstrated that a designed POSERS sample can be uniquely distinguished from any other DNA sample and experimentally validated these findings. To assess resistance against forgery, we analyzed potential forgery attack scenarios under conservative worst-case assumptions. Our results show that directly copying a POSERS library is practically infeasible: replicating the individual DNA strands would require resynthesizing millions of unique sequences, enabling the labeling of only a single forged product, an effort that is both technically prohibitive and economically irrational. Luescher et al. (2024)^7^ propose safeguarding their design from PCR amplification by incorporating short constitutive overhangs of six and seven base pairs between the randomized sequences. Although such short fragments can hinder PCR efficiency, prior research has demonstrated that amplification is still possible with comparably small primer binding sites, leaving their design vulnerable to forgery attempts via PCR.^17^ By contrast, the POSER tag can entirely omit the primer binding site from one end of the tag. Combined with its ability to track duplicates arising from amplification attempts, this gives POSER a unique and highly robust defense against PCR-based replication. Furthermore, the design cannot be reconstructed through sequence analyses, such as nucleotide distribution patterns or by systematically record all possible nucleotide combinations.

The adaptability of POSERS, enabled by *in-silico* variation of design parameters and minimal DNA sample preparation requirements based solely on DNA synthesis, allows flexible adjustment of design complexity to application-specific needs, such as product lifetime or batch size, and thus offers a secure, feasible, and future-proof solution that remains robust even in the face of ongoing advancements in DNA synthesis and sequencing technologies. In contrast to competing techniques, POSERS offer a significant advantage by eliminating the need for resource-intensive retesting of newly synthesized tags.^10,18^ This enables direct use of the tags with minimal liquid handling and without any preparation through enzymatic reactions. To demonstrate the advantages of the POSERS technology, we presented a concise comparison of its prominent competitors in the DNA tagging field (Table 1).

**Table 1:** Comparison of POSERS with Existing DNA Tagging Technologies^a^

Work	Composition of DNA sequences	Unique sequences per tag	Sequence length (bp)	DNA amount per tag (pmol)	Detection method	Anti-replication feature	Replication traceability via duplicates	PCR-impaired feature^b^	Pretest required for new tags^c^
Barka et al. **(2021)** ^7^	Combinatorial library	20	22	320	Visual	Non	No	No	Yes
**Paunescu et al. (2014)** ^25^	Fixed (single sequence)	1	113 or 238	n/a	qPCR	Non	No	No	Yes
**Doroschak et al. (2020)** ^8^	Combinatorial library	96	480	2.5	Nanopore sequencing	Non	No	No	Yes
**Luescher et al. (2024)** ^10^	Combinatorial library	Estimated hundreds	93	<0.001	Sanger electropherogram	Yes	No	Yes (non-optimal)	Yes
**Li et al. (2025)** ^18^	Combinatorial library	100 mil	21-65	6000	Visual	Yes	No	No	Yes
**POSERS (2025)**	**Combinatoria l library**	**251 k**	**200**	**<0.1**	**Illumina sequencing**	**Yes**	**Yes**	**Yes**	**No**

a Bp, base pairs; Pmol, picomole; N/A, not applicable.

b Whether the constitutive sequence incorporated into the DNA tag design, which serves as a site for PCR amplification, can be removed.

c Whether the generation of new DNA tag sequences necessitates comprehensive chemical and enzymatic validation prior to their application on the product.

We successfully demonstrated the unique strength of the POSERS strategy. Unlike existing methods, a single DNA pool synthesis step generates a large POSERS CPOL sample that allows assigning each product in a batch a unique DNA library using only five nanograms of DNA per product. The required amount of the POSERS tag is comparable to competing DNA tagging technologies.^7,8,10^ Given the continuous decrease in DNA synthesis costs,^19^ we consider this approach commercially viable. Moreover, the required amount of DNA could be further reduced by exploring alternative NGS sample preparation methods. In this study, we employed a PCR-free library preparation method. However, previous findings suggest that using other sample preparation methods incorporating PCR steps can significantly increase sequencing yield,^19^ thereby reducing the required amount of POSERS DNA and the overall cost for authentication purposes. Nonetheless, PCR amplification can introduce duplicate sequences, making careful evaluation and filtering during data analysis essential.^20^

The authentication of a POSERS tag relies on NGS library preparation and sequencing, a process that currently takes several hours and requires a trained technician. While this process is not suited for high-throughput applications at present, it is well aligned with the needs of high-value products. Importantly, sample preparation – not sequencing itself – remains the primary cost and time driver. However, this work can be outsourced to specialized service providers, making the system feasible even at larger scales if needed. Still, this entails a longer turnaround time than that of competing visual authentication methods.^7^ To further reduce cost and processing time, multiple POSERS tags can be sequenced simultaneously in a single run (multiplexing). Recent advances in NGS technology also made sample preparation more streamlined and automatable. At the time of this study, “TraxION”,^21^ a fully automated device for sample preparation and sequencing by Oxford Nanopore Technologies, was under development. Such innovations could significantly lower the barrier to broader adoption by minimizing the need for dedicated laboratory infrastructure and manual handling. These developments indicate that the challenges associated with NGS-based authentication of a POSERS tag are being actively addressed and improved, further increasing the system’s practical feasibility.

Building on our experimental validation, we demonstrated the integration of POSERS tags onto paper substrates, confirming their applicability for secure tagging in practical material systems. However, POSERS tagging application is not limited to paper. Previous studies have shown that short DNA sequences can be stably embedded in various materials, including pharmaceuticals, food products, inks, and polymers.^22–26^ These findings highlight the versatility of DNA as a molecular carrier and open up numerous potential applications for POSERS tags across various industries. Moreover, the underlying design principle of POSERS is not restricted to nucleic acids and may be adapted to other materials with precisely arranged monomers, such as proteins, and synthetic compounds such as the peptide nucleic acids (PNA),^27^ provided they offer sufficient sequence diversity and stability for effective implementation. This substrate-agnostic potential positions POSERS as a robust and adaptable anticounterfeiting strategy and opens new avenues for integrating steganographic tagging into advanced materials for supply chain security, traceability, and anticounterfeiting applications.

## Materials and Methods

### SPOL and CPOL Synthesis

To generate the oligonucleotide library, we designed a test library with a length of 40 nucleotides incorporating 20 positions with restrictions and 20 positions with no restriction. Twenty separate SPOL samples were synthesized using the IDT Pooled Oligo Pools service and subsequently pooled to form the final CPOL (AT251). Each SPOL contained a POSERS region, single-stranded sequences of 40 nucleotides, with one restricted position, which means that this position lacks two or three nucleotides. All other positions are randomly filled with an equal distribution of all four nucleotides. To facilitate PCR amplification and Illumina library preparation, 80 bases of a synthetic constitutive sequences were added on both the 5′ and 3′ ends of the designed sequence, resulting in a total oligonucleotide length 200 nucleotides.

The sequence overhangs were:

For 5′ end:

ATTGACCAACACTACTAACTTACATTTAACGTCATGCAATCTTCGAGAAGCAATGACAACGATGCCTTTGGTTATTTGAT

For the 3′ end:

ACTGAGATAGCAATATGATAAAGATGTTATTGAACGAGTGGAATGCATAGAGACAGGAATCGTCCTTGTACTGCGTCTAA

For the control sample, we used the same IDT Pooled Oligo Pools service to generate two libraries in which the 40 nucleotides from the design were replaced by fully randomized or constitutive nucleotides, flanked by the same 80 nucleotides constitutive sequences (AT252).

The sequence of all oligonucleotides used in this study are provided in Table S4.

### Double-Stranding of CPOLs

To determine the lowest required sample amount for Illumina sequencing, both the test CPOL (AT251) and the control oligo sample (AT252) were converted to double-stranded DNA using AT17 as the complementary oligo primer.

One μL from 100 pmol/μL dilution of both AT251 and AT17 was used for double stranding the CPOL library while 5 μL from 5 pmol/μL dilution of AT252 and AT250 used for the control sample. Both reactions were performed in 20 μL volume using PrimeSTAR Max DNA Polymerase (Takara Bio, Saint-Germain-en-Laye, France) under the following conditions in a thermal cycler: 98 °C for 15 s (denaturation), 55 °C for 10 s (annealing), 68 °C for 90 min (extension) for one cycle. The double-stranded CPOL was then purified using the Zymo DNA Clean & Concentrator Kit (Zymo Research, Freiburg, Germany).

### CPOL Sample Preparation and Quantification for Illumina Sequencing

The purified double-stranded CPOL sample was prepared in seven different DNA amounts, ranging from 0.01 to 25 ng, with final volumes between 1.3 to 4.2 μL. Additionally, 25 ng of the double-stranded control sample was used for sequencing.

### Extraction of CPOLs from Filter Paper

To test the extraction efficiency and influence of paper extraction on sample quality, two paper samples were prepared. For this, 25 ng of double-stranded CPOL DNA was diluted in 10 μL of ddH_2_O and applied to two MN 615 filter papers (each 2 mm*2 mm) (Machery-Nagel, Düren, Germany), which were placed inside Eppendorf tubes. The samples were left to dry for 24 h. After drying, 10 μL of ddH_2_O was added to each paper, and after several rounds of pipetting, the recovered liquid was transferred into new tubes. One sample was further purified using the Zymo DNA Clean & Concentrator Kit, while the other sample was used directly for sequencing preparation.

### PCR Sample Preparation

To analyze the impact of PCR amplification of a CPOL on the sequencing results (Figure S1), 1 ng of the double-stranded CPOL was used as a template, along with AT17 and AT250 as primers (Table S4). The PCR amplification was performed using KAPA HiFi HotStart ReadyMix PCR kit (Roche, Basel, Schweiz) under the following thermal cycling conditions: 98 °C for 45 s (initial denaturation); **25 cycles:** 98 °C for 15 s, 60 °C for 30 s, 72 °C for 30 s; 72 °C for 60 s (final extension).

PCR product was purified using the Zymo DNA Clean & Concentrator Kit (Zymo Research) and 25 ng of the purified product was used for sequencing.

### DNA Quantification

DNA concentrations for all samples were determined by DS-11 FX+ Fluorometer (DeNovix, Wilmington, DE, USA) together with the Qubit dsDNA HS Assay Kit (Invitrogen, Waltham, MA, USA).

### Library Preparation and Illumina Sequencing

For library preparation, we used the Illumina PCR-free Library Preparation Kit (Illumina Inc., San Diego, CA, USA) following the “Thermal Cycler, Low Input” protocol provided by Illumina. To enable sample identification, we applied Illumina UD Indexes to barcode each sample. The specific indexes used are listed in Table S5. Following indexing, all 11 samples were pooled into a single 1,5 mL Eppendorf tube and sent for sequencing by Microsynth AG RTL Illumina 50 Mio shotgun read pairs 2*150 service. The sequencing was run on a NovaSeq sequencer using NovaSeq v1.5 chemistry. Microsynth AG demultiplexed the sequencing result of 11 mixed samples based on the UD Indexes provided by us and delivered a separate FASTQ sequence result for each 11 samples.

### Data Analysis

The FASTQ files received from Microsynth AG were analyzed using our custom program designed to evaluate combinations of nucleotides at the restricted positions. For each data set, the program identified the nucleotide combinations present at these positions and reported the number of recorded combinations that did not follow the restrictions implemented in the design.

To analyze sequence duplications, the Clumpify tool from the BBmap package was used with the default settings for the NovaSeq sequencer (Figure S1)

## Supplementary Material



## Data Availability

The sequencing data for all 11 samples are available in the Figshare repository at 10.6084/m9.figshare.28504847. Any additional data will be made available upon reasonable request. The code from the custom analysis program used in this study is available in the Figshare repository at 10.6084/m9.figshare.28505531.v1.

## Notes

(1) Supplementary Figure 1; (2) Mathematical verification; (3) Supplementary Tables 1–5 (PDF)

## Nonstandard Abbreviations introduced in this work

SPOL: single position oligo library
CPOL: combined positions oligo library
SC: sample combination
CV: sample variety
