# Supplementary material for: POSERS: A Steganography-Driven Molecular Tagging System Using Randomized DNA Sequences for Secure Authentication
Source: ACS Omega. 2025 Oct 23;10(43):51455–65. doi: 10.1021/acsomega.5c07126 (PMC12593101; doi:10.1021/acsomega.5c07126)
Supplement: Supplementary file 1 [file ao5c07126_si_001.pdf]

Supplementary Information

POSERS: A Steganography-Driven Molecular Tagging System Using Randomized DNA Sequences for Secure Authentication

Ali Tafazoli Yazdi,[1] Peter Nejjar,[2] Lena Hochrein[1*]*

[1] University of Potsdam, Institute of Biochemistry and Biology, Faculty of Science, Potsdam, 14476, Germany.

[2] University of Potsdam, Institute of Mathematics, Faculty of Science, Potsdam, 14476, Germany.

*** Corresponding authors**

Supplementary Information

(1) Supplementary Figure 1

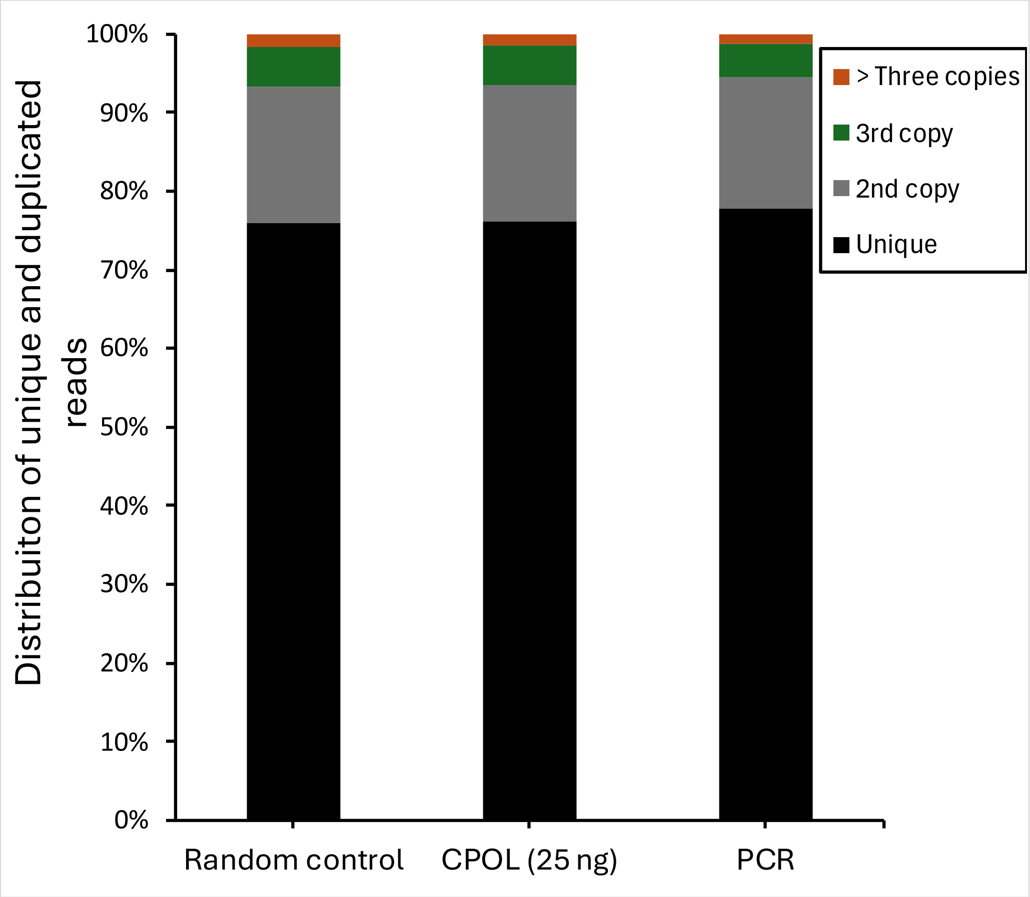

Supplementary Figure 1. Duplication report in the FASTQ sequencing results (sample 7, 8, 11 see Methods). All three library samples exhibit a similar duplication pattern. About 75% of the reads in the raw FASTQ file are unique. Approximately 17% represent the second occurrence of a unique read, while the third copy accounts for about 5% of all reads. The remaining reads correspond to the fourth and subsequent copies of a unique read in the dataset. Filtering optical duplicates from the FASTQ results using the Clumpify (bbmap) tool (https://sourceforge.net/projects/bbmap) did not help differentiate the samples based on their duplication patterns and they retain the similar pattern (see methods).

(2) Mathematical verification

Forbidden combination calculation

Here we verify the equation (1) and (2). Let $h_1, \ldots, h_K$ be the K positions picked by our library. A sequence is not authentic if at these positions we choose nucleotides $N_1, \ldots, N_K$ such that N_j is a nucleotide not allowed at position h_j in the design which has restrictions at h_j . There are $3^{K_1}2^{K_2}$ ways to choose such nucleotides.

Then in total there are $4^{L-K}3^{K_1}2^{K_2}$ sequences which are not authentic. Dividing by the total number 4^L of sequences, we get

$$\frac{4^{L-K}3^{K_1}2^{K_2}}{4^L} = \frac{3^{K_1}2^{K_2}}{4^K} = \frac{3^{K_1}2^{K_2}}{4^{K_1+K_2+K_3}} = (1)$$

As for equation (2), note that the probability of a fully randomized sequence to be authentic equals $1 - p$,

and since sequences are independent from each other, the probability that n sequences are authentic equals $(1 - p)^n$. To choose n, we thus need to solve

$$(1 - p)^n = \varepsilon$$

for n. This leads to equation (2) by taking the logarithm.

Nucleotide distribution calculation

Here we verify the equations (3) and (4). To start, note that for each SPOL we produce l sequences, so in total we produce $K\,l$ sequences. Let $q, 1 \leq q \leq L,$ be one of the K positions which has been picked. This means there is a design such that at position q, only i nucleotides are allowed, with i taking one of the values 1,2,3. By assumption, all l sequences which are produced following this design are produced with the same probability. This, in particular, implies that at position q, each of the allowed nucleotides is produced with the probability $1/i$.

Thus, within the l sequences, the expected value of the number of times we see an allowed nucleotide at position i equals l/i. Among the other $(K-1)l$ sequences, all four nucleotides appear at position q with probability $1/4$, thus the expected number of occurrences of an allowed letter at position q is $(K-1)\mathrm{l}/4$. Summing up, this shows that an allowed letter is expected to occur $(K-1)l/4 + l/i$ many times. To get to the proportion, we still need to divide by the total number Kl of sequences, yielding

$$\frac{(K-1)l/4 + l/i}{Kl} = (3).$$

The proportion equation (4) can be computed analogously, in fact one can deduce equation (4) from equation (3), by using that the proportion of all allowed nucleotides at position q equals $i \times (3)$, thus the proportion of not allowed nucleotides equals $1 - i \times (3)$, and since there are $4 - i$ not allowed nucleotides, we end up with a proportion of $\left(1 - i \times (3)\right)/(4 - i)$, which is exactly equation (4).

Total number of sequences per design

Here we explain how to justify that we may produce more than $(1-p)4^K$ sequences in total. Namely, let U be the total number of DNA sequences. Assume the forger inspects the K picked positions and has access to all U sequences. At the K positions, 4^K DNA sequences can be formed.

U should be chosen such that on average at least $p * 4^K$ from these 4^K sequences are not produced in a fully random CPOL of U sequences. We can think of each of the 4^K sequences as a coupon, and then U can be taken to be the expected number of coupons we need to collect (i.e., the number DNA sequences we need to produce) until $(1-p)4^K$ distinct coupons were obtained. This expected number equals $4^K\ (harm(4^K) - harm(p * 4^K))$ which follows from equation (2.6) in ref. 1. Here, $harm(x) = \sum_{i=1}^{x} 1/i$ denotes the harmonic numbers.

In our concrete CPOL, this would allow to produce 9.8083*$4^{20} = 1.0784 * 10^{13}$ sequences in total, an increase by almost factor 10 compared to $(1-p)\ 4^{20} = 1.0995 * 10^{12}$.

Reference

1. Levin, D. and Peres, Y. *Markov Chains and Mixing Times: Second Edition*. (AMS, 2017)

(3) Supplementary Tabels 1-5

Supplementary Table 1: Sample combination test results for the random control sample.

Full sequence of each read with restricted combination	Nucleotide found in the restricted positions.<br>Order of positions is shown as =<br>[1,4,6,7,9,11,13,15,17,19,20,22,24,26,28,31,33,35,37,39]
AGTGACGCCTTCTGCTGTCTCGTAAACATGAATACTGATC	['A', 'G', 'C', 'G', 'C', 'T', 'T', 'C', 'G', 'C', 'T', 'G', 'A', 'A', 'A', 'A', 'T', 'C', 'G', 'T']
ATAACAGTTTTTCCCTTCGGAACTTAGAGGTGCTGTGGCG	['A', 'A', 'A', 'G', 'T', 'T', 'C', 'C', 'T', 'G', 'G', 'A', 'T', 'A', 'A', 'T', 'C', 'G', 'G', 'C']
GTCACAGGTTAACTCGTTCTTGGCTCTTAGACTCAAGCAT	['G', 'A', 'A', 'G', 'T', 'A', 'C', 'C', 'T', 'C', 'T', 'G', 'C', 'C', 'T', 'A', 'T', 'A', 'G', 'A']
CTAGCTTGTTATTCCCGTTGAGGCCAGTCTTTTAGTGTTT	['C', 'G', 'T', 'T', 'T', 'A', 'T', 'C', 'G', 'T', 'G', 'G', 'C', 'A', 'T', 'T', 'T', 'G', 'G', 'T']
ACGGTCGGGATACGCGTGGTAACCTAGTAAAGTAAGGTAC	['A', 'G', 'C', 'G', 'G', 'T', 'C', 'C', 'T', 'G', 'T', 'A', 'C', 'A', 'T', 'A', 'T', 'A', 'G', 'A']
CTCAAAGATATATGGATTGACGCTTATGTGTTTTCACACG	['C', 'A', 'A', 'G', 'T', 'T', 'T', 'G', 'T', 'G', 'A', 'G', 'T', 'A', 'G', 'T', 'T', 'C', 'C', 'C']
AATGCCTTCGTTTCAGGGCACGCAACGTGGTATACAGGAG	['A', 'G', 'C', 'T', 'C', 'T', 'T', 'A', 'G', 'C', 'A', 'G', 'A', 'C', 'T', 'T', 'T', 'C', 'G', 'A']
ACTAGCTTCGTATTGGTGGATATCTACGAATCTAGTGTCT	['A', 'A', 'C', 'T', 'C', 'T', 'T', 'G', 'T', 'G', 'A', 'A', 'C', 'A', 'G', 'T', 'T', 'G', 'G', 'C']
GGGAGCGCGGTTTGGAGTGGGGTTAAAAGGAGCACGGTTT	['G', 'A', 'C', 'G', 'G', 'T', 'T', 'G', 'G', 'G', 'G', 'G', 'T', 'A', 'A', 'A', 'C', 'C', 'G', 'T']
ATGAATTCCTTCTTCGTATTGGTTACGGTCTTCCATCCAT	['A', 'A', 'T', 'T', 'C', 'T', 'T', 'C', 'T', 'T', 'T', 'G', 'T', 'C', 'G', 'T', 'C', 'A', 'C', 'A']
AACACATCGTTTTTGCTTGGTATCGCATTATTTGGGGATT	['A', 'A', 'A', 'T', 'G', 'T', 'T', 'G', 'T', 'G', 'G', 'A', 'C', 'C', 'T', 'T', 'T', 'G', 'G', 'T']
GTTGAATCTCTATAGGGACTTGGTGCAGGAATTTGGGGCT	['G', 'G', 'A', 'T', 'T', 'T', 'T', 'G', 'G', 'C', 'T', 'G', 'T', 'C', 'G', 'A', 'T', 'G', 'G', 'C']
AAGAGTTTTGACCCCTTGTAGACCGATGCATTCCCAGTTT	['A', 'A', 'T', 'T', 'T', 'A', 'C', 'C', 'T', 'T', 'A', 'A', 'C', 'A', 'G', 'T', 'C', 'C', 'G', 'T']
CTAGGTTCCTTATCAAGGGGCGGACAAATTTATTAAGGCT	['C', 'G', 'T', 'T', 'C', 'T', 'T', 'A', 'G', 'G', 'G', 'G', 'A', 'A', 'A', 'T', 'T', 'A', 'G', 'C']
ATAGAATGGGACCAGTTTGGGACTTACGAGTCCAGCGATA	['A', 'G', 'A', 'T', 'G', 'A', 'C', 'G', 'T', 'G', 'G', 'A', 'T', 'A', 'G', 'T', 'C', 'G', 'G', 'T']
GGTATATTTTTTTGGGTGACGCTGCGTTCTTTGGGCCTC	['G', 'A', 'A', 'T', 'T', 'T', 'T', 'G', 'G', 'G', 'A', 'G', 'T', 'C', 'T', 'T', 'T', 'G', 'C', 'T']
GGTGATTGCGAGTTGGTTTACACCACTGAATTCTAAGGCC	['G', 'G', 'T', 'T', 'C', 'A', 'T', 'G', 'T', 'T', 'A', 'A', 'C', 'C', 'G', 'T', 'C', 'A', 'G', 'C']
GGTATTGGTTACTTGTGTTTAATTACGACATGCGCGGATT	['G', 'A', 'T', 'G', 'T', 'A', 'T', 'G', 'G', 'T', 'T', 'A', 'T', 'C', 'A', 'T', 'C', 'C', 'G', 'T']
ACGGTATGTCAGTGAGGGCATGGTGCCTGCTTTGGTGGTT	['A', 'G', 'A', 'T', 'T', 'A', 'T', 'A', 'G', 'C', 'A', 'G', 'T', 'C', 'T', 'T', 'T', 'G', 'G', 'T']
GGGGTTTGGTTTCAGTGAGGGATAAATGGGTTTACTGGAA	['G', 'G', 'T', 'T', 'G', 'T', 'C', 'G', 'G', 'G', 'G', 'A', 'A', 'A', 'G', 'T', 'T', 'C', 'G', 'A']
CATGATTGGGAGTTAATGTATGCCGAGATTTCCTGGCATG	['C', 'G', 'T', 'T', 'G', 'A', 'T', 'A', 'T', 'T', 'A', 'G', 'C', 'A', 'A', 'T', 'C', 'G', 'C', 'T']
AGTGCAGTTTAGCGAGGATATAGTGCTTTAATTGAAGCCG	['A', 'G', 'A', 'G', 'T', 'A', 'C', 'A', 'G', 'T', 'A', 'A', 'T', 'C', 'T', 'A', 'T', 'A', 'G', 'C']
GTAGTTTTTGAGTCCTGTGAGAGTAAAAGATGTCGTGGCG	['G', 'G', 'T', 'T', 'T', 'A', 'T', 'C', 'G', 'G', 'A', 'A', 'T', 'A', 'A', 'T', 'T', 'G', 'G', 'C']
CAGGGAGTCAAGCGCCGTGTAGCATCGTAGTACTGTGGAG	['C', 'G', 'A', 'G', 'C', 'A', 'C', 'C', 'G', 'G', 'T', 'G', 'A', 'C', 'T', 'T', 'C', 'G', 'G', 'A']
ACTGACGTGGATTAGTTGCGGGCTAAGGAGAATTCCGGTT	['A', 'G', 'C', 'G', 'G', 'A', 'T', 'G', 'T', 'C', 'G', 'G', 'T', 'A', 'G', 'A', 'T', 'C', 'G', 'T']
GTCGTCGTGCTTCGGAGGGTAATTCCGTAATGTGATGGCT	['G', 'G', 'C', 'G', 'G', 'T', 'C', 'G', 'G', 'G', 'T', 'A', 'T', 'C', 'T', 'T', 'T', 'A', 'G', 'C']
GGTATCTCTATATGACTGTTTGATTACTTGTGCAGTGTAG	['G', 'A', 'C', 'T', 'T', 'T', 'T', 'A', 'T', 'T', 'T', 'G', 'T', 'A', 'T', 'T', 'C', 'G', 'G', 'A']
GGTGCTTGGGACCCACGATAGGTCAAGGTGTTTTGGCATG	['G', 'G', 'T', 'T', 'G', 'A', 'C', 'A', 'G', 'T', 'A', 'G', 'C', 'A', 'G', 'T', 'T', 'G', 'C', 'T']
CCAACCTTGTTTCGCGGTTGCATAACTTAGTCTAGTGCAA	['C', 'A', 'C', 'T', 'G', 'T', 'C', 'C', 'G', 'T', 'G', 'A', 'A', 'C', 'T', 'T', 'T', 'G', 'G', 'A']

Supplementary Table 2: Sample variety test result for CPOL sample (25 ng).

SPOL number	List of sequences found for each SPOL	Number of reads per SPOL	List of nucleotides found in each position	Predicted design	Missing nucleotide in the predicted design that is found in the CPOL sample
1	['TTGATAGATAAGCCAGGATAGGTTGATACCAGTCACCTCC', 'TGTGACGGTTTTTGCGGACATAGTGAGATTTGTGGGGGAC', 'TTGATCGTGTAGTAGGGATATGTCGATTGATTTAAGGGTG', 'TGAAGTGTGGATTACATGTTAGGAAATGTGTCTGGAGGTA', 'TGGGCCGCTGTGCCCAGAGAGGATGACAACTGCGGTCACT', 'TGGGACGTTTTGTTGTTCCGGGATCCCATGAATTCGGTCT', 'TTAGCTGTTAAACGGTGGGGTATAGCTGAATGCGAGGGCA', 'TGTATTTTGCTTCGGGTAGTAGTATCATCTAACTATGACG', 'TTCGTAGTTGATCAGTTCGGTAGTACCTCGTGCTCTGATG']	9	['T', 'T', 'T', 'T', 'T', 'T', 'T', 'T', 'T']	T	
4	['GTACTTGTTGATCACAGGCTAAGTACAAGCATCGGACGCC', 'ACCTTTTGCTACCCATGGGTGGACACGAGGTGTGCCGAAA', 'CAATAATAGAAGTGGCGGGAAGGTTAGACGAACTCAGCTA', 'GGGTTCGCGGTGTCATGTGGAATTGCCGATATTACACTAA', 'GTTCACGATTAATCATGGTTTAGCGACTCGTGTGCACTAC']	5	['C', 'T', 'T', 'T', 'C']	T	C
6	['GGGACGTGTGTTTTGGGTGTGATACAGTTGTCTAGGGGCA', 'GGGGAGGTGTTACAATTTTGGATAGCGGGGAGCGCTCGCG', 'AATATGTCGATGCGGTTGGGCGACTAGGTTTATGGTGCAG']	3	['G', 'G', 'G']	G	
7	['CATATTAAGTTACGCGTGTTAGGACACTTGTGTGGACGTT', 'GCCGCTCGTTTGTTATTTCATGGTCAGATGTATTCACGAT', 'AGAATTCGTTTCTGCTGTGTGGGCTACGAGTCCGGACCAG', 'CTAAGTAACGTGTCATGGTTGATTACTTCATTTTCTGAAC']	4	['A', 'C', 'C', 'A']	A	C
9	['GGGGGAGGAATCTAGTGCGGAGGTACTGAATGCTGAGACG', 'AAGGGATAACTCTCGGTCTAAGTTAAAACTAATCACGGCG', 'CTCGTATTATTATAAGTAGTAGTCAACTTATCCACTCGTA', 'AATAGCTTATTTTGGGTTGAGGTTAAAAAGACTGAGGCAA', 'CGAAGTGGAGAATTGGGCTAGGAACACAAATGCCAGGTTA', 'GATAGTTAAGTTTTCCGTGTTGGTACGTATTGTAGAGGTA', 'CGTGCTGTAGTCCCCGTGCGGAACCCAAGGATCGCCCAAT', 'GTAGGAGTAATGCAAAGGTGGGCAGACAGAATCAAGGGTA']	8	['A', 'A', 'A', 'A', 'A', 'A', 'A', 'A']	A	
11	['ACTGGTTCTCGGTAGTGTGTTGTACCAACGTGTACAGATG', 'CTGATTTGGAGTTTGGTCGGGAGTGAAATTAACGGTGATT', 'ACGAATGGCGCACTCGGTGAGGCCTATAGCAGTCCTGTTT']	3	['G', 'G', 'C']	G	C
13	['CGGGTTGTCTTCGCAGTAGGCAGAAACGGAATCCGCGATT', 'GGGGACGTGGTTGGAGGTTAAACATACGAGTTTCCACGTC', 'GTCGGTGTTGTCGGGTGTGAGGTTAATTTGAATCGGCACG', 'GATATTTGGGTGGCGCGACGGGTTCAATACTGTAAACGAG', 'CTCATCGGTATTATCGTTCATGGATACTACTTTGGGCTTT', 'GACGTTGATGTCAGGGGTGGCGGCTCCTAGTATCGCGTTC', 'GTGGGTTGGAATGTATTATTAGACCCGATTTGTTGTCGTA', 'ACAGTATTTGATGAAGGGTGCAATCACACGAACGGTCGCC']	8	['G', 'G', 'G', 'G', 'A', 'A', 'G', 'G']	G	A
15	[]	0	[]	T	
17	['AGGATATTGGAGCGACCGGTGAGTTCGGTTTCTAGGCGAG', 'CAGGGATCTATCCTGCACGATGTCAAGGGGAGCTGGCACG', 'CCGGCAGATAAGCGGCATGGCAGAGATTTATGCGCGGCAC']	3	['C', 'A', 'A']	A	C
19	['CCGGGCTTTGACTTGATGAGGGACAACAAATGTCATGCTT', 'GGCAATTATGTGCACCTAATTGAATCTTGCATTGGGGATC', 'CCTACCTACGTGTGAATTAATAGTGCGTAGTTTTAGGTTC', 'ATGAGAGTTCAACAATTAATAGTTCCAGATTCTAACCGAT', 'CATATAGTTGACTAGCGTAGCGGTTCGGGCATTGATGTCT', 'CTGGGTTCGCAACTGCGAATTGTCGCCTAGTATTCCGTTG', 'CCGGTCTTCATGCTGCTCATGAATGATGTGTCTTCTCGAT']	7	['A', 'A', 'A', 'A', 'A', 'A', 'A']	A	
20	['CAAGTTTAGGTGTTCTTGGCTGGTAACGGCAATAGGGTCA']	1	['C']	C	
22	['CATATTGCTCAATACGGGTTTTTCGAGGGCTACCAACTAA', 'GTAAGCTAGATCTAATTCGATTCCCACTGGAGTCCGCTTA', 'GGGGGAGCTCTGTTACGCTAACTTACTAAGTTTAATCTTG', 'ATTAGTGTTATCCCCTTTTAGTTAACAGTTTTTAGTCGAT', 'GACGGTTATGTGCACGTCGGATGCAAAAAGTCCTATGCTG']	5	['T', 'T', 'C', 'T', 'T']	N	
24	['GGGGCTTCGCTCTCGCTCTTGAAGTATTACTTCGGGGTAG', 'GTGGGCGATGATTAACTTCGTGTGGCTAGCTTTTGGGTTT', 'GGAGGTTAGTATCTGTGGTGGGAGGAGTCCACTGCTGTAT']	3	['G', 'G', 'G']	G	
26	['CACGTATCCTTTCGCATGCTGGATGGTATTTGCCCACACA', 'ATCATCGCCAATTTGGTGTTGGGCTTTATTTGCGCCCCAA', 'CCGATCTTTGACTCACGGCGCGAAGTAAATTGCAGACGTG', 'ATGAGCGTTAAATGATGGCGTGAATGGAACTTTGCAGGTT', 'CCTGGATGTATTTGGGTGCGGATTTTGATTACTTACGGCG', 'CGCAGTGGTGTTCACGGAGAAATTTGTGGCAACCGAGTTC', 'ACTAGATTGTTTCTCCTTGTAGGCCGCTGATACGGTGGTG']	7	['G', 'T', 'T', 'G', 'T', 'G', 'G']	N	
28	['CAAGGCGAGCTGTCACGACAAACCGACCAGTTTGCAGACC', 'ACAGGCTATGTGCAAGTATAGGGTTCCCGTTGCGCAGGCG', 'GGAGTCTTTCATCAATGTCACGGCACGCCGACCTACCGCC', 'GTTAATTACGAGTGCGGTCTGGTTTCGCTTTACGGGGCTT', 'CCCAGCTACCAGTCGGTACATGACTAACGGTTCAGTCTCA', 'GGTGTTGATTTTTGGTTGTACGTTTAGCGTTTTTCTCTTG', 'GGGATTTTTGTCCAGGTGGTGGGCTATCGTATTTGGGGCG', 'GGGGGTTGTGTTTGGGTATTGGACAACCAGTTTTACGTAT']	8	['C', 'C', 'C', 'C', 'C', 'C', 'C', 'C']	C	
31	['GTGGAAGCTATTCGCGGCCTAAGTACTGTGCGTGACGCTC', 'CCCAATGATGTGCTCGGGTGTGGACCCTTTGTTTCCCCAG', 'CCTGTTGCGTACCCGGTTTTGAGTACCTTTGTTCGAGCTC', 'GTGATTTATAAGTTAGGGTGAGTCGCTTTTGGCTATGATG', 'CGAGGCTTTGATTGAAGGTGTAAATCAAATGCCGAACGCA', 'GCTGGCGTGTTCCGCCGCGGCGTTCCGATTGTCGAGCGTC']	6	['C', 'G', 'G', 'G', 'G', 'G']	N	
33	['GGGGGTGAGGAGTGGGTTTGGAGTAAATTTTAAACGCGAG', 'AACACCTCCGTCCAATTATATGGTCCTATTTTGTCCCCTG', 'CGGGCAGTGGATTAATTTGTGAAAACAGGTTTGCAGGTTC', 'AGAGAATGCCAGCCGGTACTCGGCGATAGGTCATGTGGAG', 'AGAGAATGTGAGCCACGCTTTATCGCCTGATGAGGGGCCT']	5	['A', 'G', 'G', 'A', 'A']	G	
35	['CGTGCATCGATATTGTGTGAAACCTCGATTATTTTTGCCC', 'AGGGGCGTCCTTCGATTTTTGGGTCAGTTTAATATGCTTC', 'CTAAATGAGGAGCGCGTGGACACCGATGAATGTGTGCTCG', 'AAAGCCGCCTTCTGCGTTGGGGGTCCGAGCTTTGTGGAAG', 'GGGAGAGTCATCCGAGTAGATGGAGAGTTGTTTATACCTA', 'GTCGAATATGTATTCGTGTTTATATCGGACTGCGTTGCCG']	6	['T', 'T', 'T', 'T', 'T', 'T']	T	
37	['CGGGGCGATTAGCCGTGAGGAAACGACGTTTGTGCGAGCG', 'CAGATATTTAATTGGGGACATATATCTGGCTGTACAATTA', 'GGGGCATGTAAGTCGGTGTGAGTTAATGCAAACCGGAGTG', 'GTTAGAGTGGAGTACCTCGGCATTCCCGGAAGTGGGTTTG', 'ACCAATTCGATTTAAGGTGTGGCTCAAGTTTACGGCTTTG', 'GCGAACTCTTTTTGAGGCGTTGTCACTTGCTCTTATAGTG', 'CCTGGTGTCTTTCGCTGTGATGGATCCGGCAACGAATTCG', 'ACCAAATGTGTTTGCCGGCGGGGTACGTATATCTGTTACT', 'CGAAATTCTGTATGGTTCTTGAAATCGGGGTTCCCAAGTT', 'GGAGGCTTGATATGGTTCTGAAAATAGAGTAATGCTTTTA']	10	['A', 'A', 'A', 'T', 'T', 'A', 'T', 'T', 'A', 'T']	T	A
39	['GTGGTATCTTTGTGCGTACGCGTCGCTTCTTTCGGACAGT', 'GTTACAGACTACTTGGGTTGTGGTCCTTCGAGCGGTGGGC', 'GCCGAATTTTAGTAATGTTGAGATCATTACTGCTCACCGG']	3	['G', 'G', 'G']	G	

Supplementary Table 3: Potential prediction of a POSERS library by a forger.

Prediction description:

First, the average percentage of nucleotide distribution across all positions is calculated (Avg). To establish a threshold, 1.25% is added to each average value. Any nucleotide with a distribution percentage exceeding this threshold is considered a nucleotide at a restricted position. Nucleotides surpassing the threshold are marked in blue. For each position, if no nucleotide is marked blue, the position is considered unrestricted. Conversely, all positions with blue-colored nucleotides are considered restricted and the respective nucleotides in those positions are considered the allowed nucleotides at that position. The results of this prediction are color-coded based on comparison with the original design.

Design positions	Original design	Percentage of nucleotide distribution in each position				Predicted design
		A	T	C	G	
1	T	19.91	33.51	17.78	28.79	T
2	N	21.13	30.24	18.71	29.92	N
3	N	20.98	29.87	18.72	30.43	N
4	Y	19.78	31.57	19.8	28.85	T
5	N	21.03	29.51	18.59	30.87	N
6	G	19.9	28.44	17.72	33.94	G
7	M	22.34	28.4	19.87	29.39	A
8	N	20.83	29.76	18.78	30.63	N
9	A	25.27	28.2	17.67	28.86	A
10	N	20.89	29.25	18.95	30.9	N
11	S	19.56	27.74	19.94	32.76	G
12	N	20.89	29.5	18.71	30.9	N
13	R	21.59	28.33	17.74	32.34	G
14	N	20.8	29.7	18.5	31	N
15	T	19.61	32.95	17.87	29.57	T
16	N	20.74	29.74	18.6	30.92	N
17	M	22.14	28.31	19.96	29.59	A
18	N	20.69	29.93	18.64	30.74	N
19	A	24.87	28.22	17.6	29.31	A
20	C	19.98	28.4	21.7	29.92	C
21	N	20.65	29.88	18.56	30.9	N
22	Y	20	30.52	19.35	30.12	N
23	N	20.58	29.72	18.71	30.99	N
24	G	19.64	28.21	17.71	34.45	G
25	N	20.52	29.67	18.74	31.07	N
26	K	19.74	30.41	18.03	31.83	N
27	N	20.5	29.67	18.85	30.98	N

28	C	18.91	27.37	24.94	28.78	C
29	N	20.64	29.54	18.9	30.92	N
30	N	20.51	29.74	18.82	30.93	N
31	S	19.81	28.52	19.55	32.12	N
32	N	20.39	29.67	18.71	31.23	N
33	R	21.38	28.07	17.78	32.77	G
34	N	20.28	29.84	18.59	31.29	N
35	T	19.4	32.68	17.92	30	T
36	N	20.26	29.73	18.76	31.25	N
37	W	21.23	31.6	17.64	29.53	T
38	N	19.91	29.68	18.77	31.65	N
39	G	18.97	27.33	17.25	36.46	G
40	N	20.84	29.23	19.76	30.17	N
**Avg**		**20.68**	**29.57**	**18.83**	**30.93**	
**Threshold for a restricted nucleotide**		**21.93**	**30.82**	**20.08**	**32.18**	

Color	Definition
(green)	Correctly predicted restricted position
(light green)	Correctly predicted non-restricted position
(light pink)	Type b false predicted position
(dark orange)	Type a.2 false predicted position

Abbreviation	Nucleotides
Y	C or T
M	A or C
S	C or G
K	T or G
R	A or G
W	A or T

Supplementary Table 4: List of oligonucleotides used in this study.

Oligo name	SPOL	Sequence
AT251(CPOL)	SPOL1	GATTGACCAACACTACTAACTTACATTTAACGTCATGCAATCTTCGAGAAGCAATGACAACGATGCCTTTGGTTATTTGATNNNNNNNNNNNNNNNNNNNNNNNN<br>NNNNNNNNNNNNNNNNNACTGAGATAGCAATATGATAAAGATGTTATTGAACGAGTGGAATGCATAGAGACAGGAATCGTCCTTGTACTGCGTCTAA
	SPOL2	GATTGACCAACACTACTAACTTACATTTAACGTCATGCAATCTTCGAGAAGCAATGACAACGATGCCTTTGGTTATTTGANNNYNNNNNNNNNNNNNNNNNNNN<br>NNNNNNNNNNNNNNNNNNACTGAGATAGCAATATGATAAAGATGTTATTGAACGAGTGGAATGCATAGAGACAGGAATCGTCCTTGTACTGCGTCTAA
	SPOL3	GATTGACCAACACTACTAACTTACATTTAACGTCATGCAATCTTCGAGAAGCAATGACAACGATGCCTTTGGTTATTTGANNNNNGNNNNNNNNNNNNNNNNNN<br>NNNNNNNNNNNNNNNNNNACTGAGATAGCAATATGATAAAGATGTTATTGAACGAGTGGAATGCATAGAGACAGGAATCGTCCTTGTACTGCGTCTAA
	SPOL4	GATTGACCAACACTACTAACTTACATTTAACGTCATGCAATCTTCGAGAAGCAATGACAACGATGCCTTTGGTTATTTGANNNNNNNMNNNNNNNNNNNNNNNN<br>NNNNNNNNNNNNNNNNNNACTGAGATAGCAATATGATAAAGATGTTATTGAACGAGTGGAATGCATAGAGACAGGAATCGTCCTTGTACTGCGTCTAA
	SPOL5	GATTGACCAACACTACTAACTTACATTTAACGTCATGCAATCTTCGAGAAGCAATGACAACGATGCCTTTGGTTATTTGANNNNNNNNNANNNNNNNNNNNNNNN<br>NNNNNNNNNNNNNNNNNNACTGAGATAGCAATATGATAAAGATGTTATTGAACGAGTGGAATGCATAGAGACAGGAATCGTCCTTGTACTGCGTCTAA
	SPOL6	GATTGACCAACACTACTAACTTACATTTAACGTCATGCAATCTTCGAGAAGCAATGACAACGATGCCTTTGGTTATTTGANNNNNNNNNNNSNNNNNNNNNNNNN<br>NNNNNNNNNNNNNNNNNACTGAGATAGCAATATGATAAAGATGTTATTGAACGAGTGGAATGCATAGAGACAGGAATCGTCCTTGTACTGCGTCTAA
	SPOL7	GATTGACCAACACTACTAACTTACATTTAACGTCATGCAATCTTCGAGAAGCAATGACAACGATGCCTTTGGTTATTTGANNNNNNNNNNNNNRNNNNNNNNNN<br>NNNNNNNNNNNNNNNNNNACTGAGATAGCAATATGATAAAGATGTTATTGAACGAGTGGAATGCATAGAGACAGGAATCGTCCTTGTACTGCGTCTAA
	SPOL8	GATTGACCAACACTACTAACTTACATTTAACGTCATGCAATCTTCGAGAAGCAATGACAACGATGCCTTTGGTTATTTGANNNNNNNNNNNNNNNTNNNNNNNNN<br>NNNNNNNNNNNNNNNNNACTGAGATAGCAATATGATAAAGATGTTATTGAACGAGTGGAATGCATAGAGACAGGAATCGTCCTTGTACTGCGTCTAA
	SPOL9	GATTGACCAACACTACTAACTTACATTTAACGTCATGCAATCTTCGAGAAGCAATGACAACGATGCCTTTGGTTATTTGANNNNNNNNNNNNNNNNNNMNNNNN<br>NNNNNNNNNNNNNNNNNNACTGAGATAGCAATATGATAAAGATGTTATTGAACGAGTGGAATGCATAGAGACAGGAATCGTCCTTGTACTGCGTCTAA
	SPOL10	GATTGACCAACACTACTAACTTACATTTAACGTCATGCAATCTTCGAGAAGCAATGACAACGATGCCTTTGGTTATTTGANNNNNNNNNNNNNNNNNNNNANNN<br>NNNNNNNNNNNNNNNNNNACTGAGATAGCAATATGATAAAGATGTTATTGAACGAGTGGAATGCATAGAGACAGGAATCGTCCTTGTACTGCGTCTAA
	SPOL11	GATTGACCAACACTACTAACTTACATTTAACGTCATGCAATCTTCGAGAAGCAATGACAACGATGCCTTTGGTTATTTGANNNNNNNNNNNNNNNNNNNNNCNN<br>NNNNNNNNNNNNNNNNNNACTGAGATAGCAATATGATAAAGATGTTATTGAACGAGTGGAATGCATAGAGACAGGAATCGTCCTTGTACTGCGTCTAA
	SPOL12	GATTGACCAACACTACTAACTTACATTTAACGTCATGCAATCTTCGAGAAGCAATGACAACGATGCCTTTGGTTATTTGANNNNNNNNNNNNNNNNNNNNNNNY<br>NNNNNNNNNNNNNNNNNNACTGAGATAGCAATATGATAAAGATGTTATTGAACGAGTGGAATGCATAGAGACAGGAATCGTCCTTGTACTGCGTCTAA
	SPOL13	GATTGACCAACACTACTAACTTACATTTAACGTCATGCAATCTTCGAGAAGCAATGACAACGATGCCTTTGGTTATTTGANNNNNNNNNNNNNNNNNNNNNNNN<br>NGNNNNNNNNNNNNNNNNACTGAGATAGCAATATGATAAAGATGTTATTGAACGAGTGGAATGCATAGAGACAGGAATCGTCCTTGTACTGCGTCTAA
	SPOL14	GATTGACCAACACTACTAACTTACATTTAACGTCATGCAATCTTCGAGAAGCAATGACAACGATGCCTTTGGTTATTTGANNNNNNNNNNNNNNNNNNNNNNNN<br>NNNKNNNNNNNNNNNNNNACTGAGATAGCAATATGATAAAGATGTTATTGAACGAGTGGAATGCATAGAGACAGGAATCGTCCTTGTACTGCGTCTAA
	SPOL15	GATTGACCAACACTACTAACTTACATTTAACGTCATGCAATCTTCGAGAAGCAATGACAACGATGCCTTTGGTTATTTGANNNNNNNNNNNNNNNNNNNNNNNN<br>NNNNNCNNNNNNNNNNNNACTGAGATAGCAATATGATAAAGATGTTATTGAACGAGTGGAATGCATAGAGACAGGAATCGTCCTTGTACTGCGTCTAA
	SPOL16	GATTGACCAACACTACTAACTTACATTTAACGTCATGCAATCTTCGAGAAGCAATGACAACGATGCCTTTGGTTATTTGANNNNNNNNNNNNNNNNNNNNNNNN<br>NNNNNNNNSNNNNNNNNNACTGAGATAGCAATATGATAAAGATGTTATTGAACGAGTGGAATGCATAGAGACAGGAATCGTCCTTGTACTGCGTCTAA
	SPOL17	GATTGACCAACACTACTAACTTACATTTAACGTCATGCAATCTTCGAGAAGCAATGACAACGATGCCTTTGGTTATTTGANNNNNNNNNNNNNNNNNNNNNNNN<br>NNNNNNNNNNRNNNNNNNACTGAGATAGCAATATGATAAAGATGTTATTGAACGAGTGGAATGCATAGAGACAGGAATCGTCCTTGTACTGCGTCTAA
	SPOL18	GATTGACCAACACTACTAACTTACATTTAACGTCATGCAATCTTCGAGAAGCAATGACAACGATGCCTTTGGTTATTTGANNNNNNNNNNNNNNNNNNNNNNNN<br>NNNNNNNNNNNNTNNNNNACTGAGATAGCAATATGATAAAGATGTTATTGAACGAGTGGAATGCATAGAGACAGGAATCGTCCTTGTACTGCGTCTAA
	SPOL19	GATTGACCAACACTACTAACTTACATTTAACGTCATGCAATCTTCGAGAAGCAATGACAACGATGCCTTTGGTTATTTGANNNNNNNNNNNNNNNNNNNNNNNN<br>NNNNNNNNNNNNNNWNNNACTGAGATAGCAATATGATAAAGATGTTATTGAACGAGTGGAATGCATAGAGACAGGAATCGTCCTTGTACTGCGTCTAA
	SPOL20	GATTGACCAACACTACTAACTTACATTTAACGTCATGCAATCTTCGAGAAGCAATGACAACGATGCCTTTGGTTATTTGANNNNNNNNNNNNNNNNNNNNNNNN<br>NNNNNNNNNNNNNNNNGNACTGAGATAGCAATATGATAAAGATGTTATTGAACGAGTGGAATGCATAGAGACAGGAATCGTCCTTGTACTGCGTCTAA
AT252		GATTGACCAACACTACTAACTTACATTTAACGTCATGCAATCTTCGAGAAGCAATGACAACGATGCCTTTGGTTATTTGAACACCTCTACCGGCGACTCTCTCG<br>AAATTTTTCTTAACGGACTGAGATAGCAATATGATAAAGATGTTATTGAACGAGTGGAATGCATAGAGACAGGAATCGTCCTTGTACTGCGTCTAA
		GATTGACCAACACTACTAACTTACATTTAACGTCATGCAATCTTCGAGAAGCAATGACAACGATGCCTTTGGTTATTTGANNNNNNNNNNNNNNNNNNNNNNNN<br>NNNNNNNNNNNNNNNNNNACTGAGATAGCAATATGATAAAGATGTTATTGAACGAGTGGAATGCATAGAGACAGGAATCGTCCTTGTACTGCGTCTAA
AT17		TTAGACGCAGTACAAGGACG
AT250		GATTGACCAACACTACTAACTTAC

Supplementary Table 5: Information about library preparation and Illumina sequencing.

Sample number	Sample name	DNA amount used for sequencing library preparation (ng)	Original pool library	Original pool library
1	Double stranded CPOL	0.01	A251	UDP0017
2	Double stranded CPOL	0.1	A251	UDP0025
3	Double stranded CPOL	0.5	A251	UDP0049
4	Double stranded CPOL	1	A251	UDP0057
5	Double stranded CPOL	5	A251	UDP0065
6	Double stranded CPOL	10	A251	UDP0073V3
7	Double stranded CPOL	25	A251	UDP0081
8	PCR amlified from CPOL	25	A251	UDP0089
9	Double stranded CPOL applied on paper (not purified)	25	A251	UDP0010
10	Double stranded CPOL applied on paper (purified)	25	A251	UDP0026
11	Double stranded control sample	25	A252	UDP0034
